# Isolation of soybean protein P34 from oil bodies using hydrophobic interaction chromatography

**DOI:** 10.1186/1472-6750-8-27

**Published:** 2008-03-11

**Authors:** Eva Sewekow, Lars Christian Keßler, Andreas Seidel-Morgenstern, Hermann-Josef Rothkötter

**Affiliations:** 1 Institute of Anatomy, Medical Faculty, Otto-von-Guericke University Magdeburg, Leipziger Str. 44, 39120 Magdeburg, Germany; 2 Max Planck Institute for Dynamics of Complex Technical Systems, Sandtorstr.1, 39106 Magdeburg, Germany; 3 Institute of Process Engineering, Otto von Guericke University, Universitätsplatz 2, D-39106 Magdeburg, Germany

## Abstract

**Background:**

Soybeans play a prominent role in allergologic research due to the high incidence of allergic reactions. For detailed studies on specific proteins it is necessary to have access to a large amount of pure substance.

**Results:**

In this contribution, a method for purifying soybean (*Glycine max*) protein P34 (also called Gly m Bd 30 K or Gly m 1) using hydrophobic interaction chromatography is presented. After screening experiments using 1 mL HiTrap columns, Butyl Sepharose 4 FF was selected for further systematic investigations. With this stationary phase, suitable operation conditions for two-step gradient elution using ammonium sulphate were determined experimentally. The separation conditions obtained in a small column could be scaled up successfully to column volumes of 7.5 and 75 mL, allowing for high product purities of almost 100% with a yield of 27% for the chromatographic separation step. Conditions could be simplified further using a onestep gradient, which gave comparable purification in a shorter process time. The identity of the purified protein was verified using in-gel digestion and mass spectrometry as well as immunological techniques.

**Conclusion:**

With the technique presented it is possible to produce, within a short timeframe, pure P34, suitable for further studies where an example antigen is needed.

## Background

In recent years, soybeans were identified as one of the main sources of allergic reactions in humans [1]. The incidence of adverse reactions to food antigens is especially high in children (2–8%, compared to adults with 1–2%) [2]. Since the spectrum varies from clinical to systemic anaphylactic symptoms [2], there is a need to develop models analysing how food antigens reach the immune cells eliciting these allergenic reactions. Especially food proteins which cause adverse reactions only in some patients are of interest. As an increasing number of food products is enriched with plant proteins due to their emulsifying properties, simple production and good digestibility, detailed studies on their allergenic potential are necessary.

In this contribution, a new purification procedure for P34 (also called Gly m Bd 30 K or Gly m 1) was developed. The protein was discovered to be the main allergen for soybean sensitive humans [3]. Soybean protein P34 is a monomeric insoluble glycoprotein with an isoelectric point of 4.5 [4,5] and an amino acid based calculated mass of 28.643 Da according to the Informall database [6], representing 2–3% of total soybean protein [7]. In its glycosylated form, the mass will be slightly larger, resulting in a band of ~32 kDa in non-reduced SDS PAGE gels [8]. As a thiol protease, it belongs to the papain superfamily. Due to an absence of catalytic cysteine it exhibits no enzymatic function [7]. In disrupted plant cells, P34 associates to soybean oil bodies, but has no membrane insertion region and is stored in storage vacuoles of soybean cotyledons [7,8]. After translation, P34 loses the pre- and pro-protein region containing one glycosylation and during seedling growth a basic decapeptide is removed [7-9]. P34 attaches the 7S globulin fraction due to disulfide bridges [10] and exists as a dimer of 58 kDa in non-reduced SDS PAGE gels [9].

The protein can be extracted from washed soybean oil body pads with 0.1 M sodium carbonate, thus allowing for a simple pre-purification [8,11]. Alternatively, P34 could be produced as a recombinant protein in *E. coli *[12]. A subsequent purification, however, is in any case necessary.

A different approach to purifying P34, based on globulin fractionation [13,14], was proposed by the group of Ogawa in 1993 [4]. There, protein P34 was isolated from the 7S globulin fraction with a multi-step process, including a chromatographic separation using Con A Sepharose.

**Figure 1:**
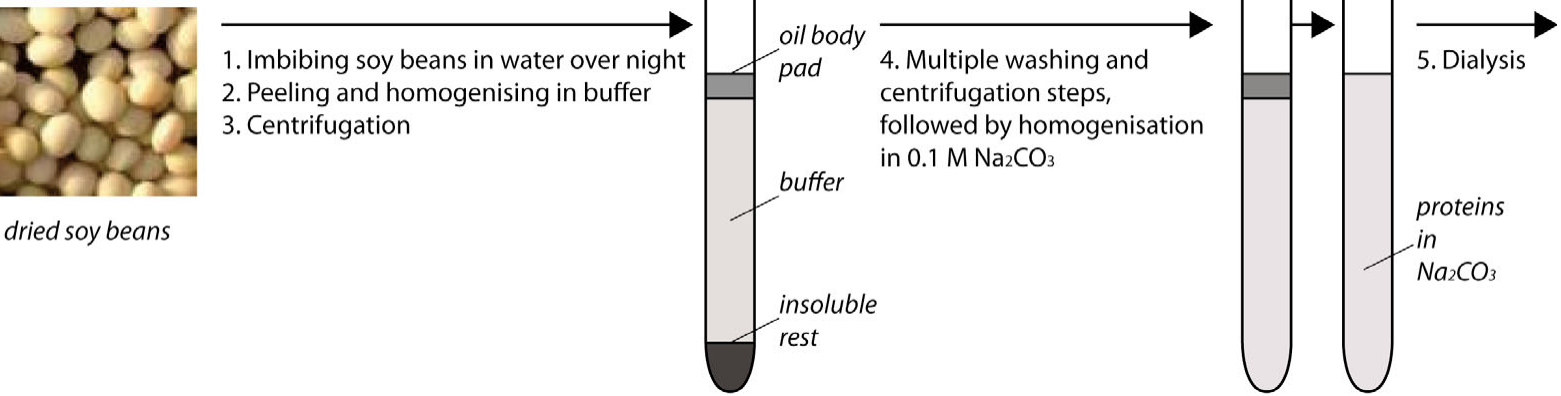
Schematic representation of soybean feedstock preparation according to [11].

In this work, a new approach for isolating pure and native P34 from soybeans for allergologic studies is presented. Soy proteins are extracted from soybean oil bodies using sodium carbonate buffer, similar to the procedure described by Herman [11]. This procedure is shown schematically in Fig. 1. Subsequently, the solution is diafiltrated and P34 is purified using hydrophobic interaction chromatography (HIC). Over the last years, HIC chromatography has experienced an increase in attention as an alternative to ion exchange chromatography for the purification of proteins [15-18]. HIC chromatography utilises hydrophobic ligands to exploit differences in protein hydrophobicity for separation. In contrast to reversed phase (RP) chromatography, however, where similar ligand types are used, adsorption in HIC is salt mediated. The addition of an anti-chaotropic salt promotes hydrophobic interactions, thereby leading to protein adsorption. Desorption can be achieved be decreasing the salt concentration in the liquid phase. The exact retention mechanism of HIC, however, is still not fully understood. Although several models have been proposed, none was accepted generally [17]. As a further distinction from RP chromatography, it is possible to use aqueous buffer systems, which is beneficial for protein stability. With this simple single-step technique it was possible to obtain high purity in a considerably shorter process time than with the other reported purification process [4].

## Results and Discussion

### Preparation of soybean protein feedstock for chromatographic separations

**Figure 2:**
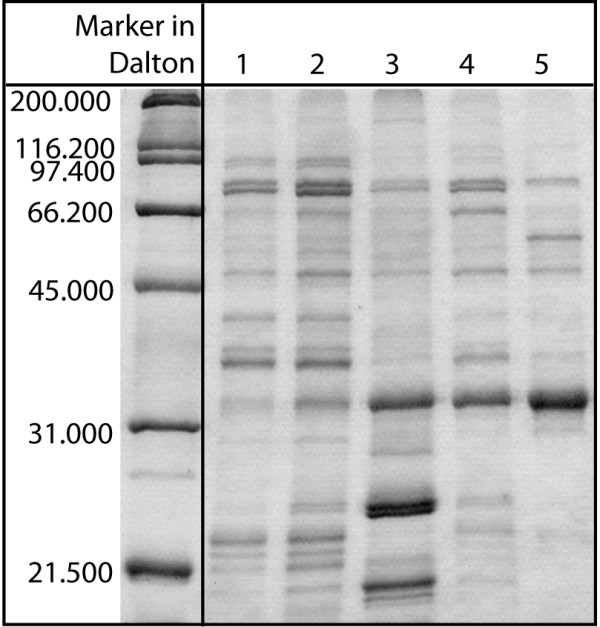
**Gel-electrophoretic analysis of protein samples from different stages of the process**. Different protein samples collected during the preparation of the soybean feedstock were loaded on a Coomassie-stained polyacrylamide gel (2 μg each lane): 1 homogenate before centrifugation, 2 oil body after the first centrifugation step, 3 oil body after the last washing step with sodium carbonate, 4 sodium carbonate supernatant, 5 dialysed protein mixture before chromatographic separation.

Protein preparation was performed as described in the Methods section, following the protocol developed by Herman [11]. This established process scheme was applied to generate the feedstock that will be used in chromatography. During the last step of the centrifugation protocol, P34 diffused selectively into 0.1 M sodium carbonate buffer. Almost 77% P34 purity could be reached after this procedure. In Fig. 2, the results of a gel-electrophoretic analysis of protein samples from different stages of the process are compared. Protein P34 is represented by a band at a mass of about 32 kDa. The P34 content of each sample was determined using densitometric techniques. From lane 1 to lane 5, the P34 fraction was calculated to be 7% in the homogenate before centrifugation (lane 1), 15% in the oil body after the first centrifugation step (lane 2), 22% in the oil body after the last washing step with sodium carbonate (lane 3), 41% in the sodium carbonate supernatant (lane 4) and 77% in the dialysed protein mixture before the chromatographic separation (lane 5), compared to the total protein content of each lane (2 μg). The mixture shown in lane 5 was used as starting material for HIC chromatography.

### Selection of suitable chromatographic conditions

During process development, a wide range of possible purification strategies was tested. However, with neither size exclusion, lectin-based affinity, cation exchange nor anion exchange chromatography was it possible to meet purity requirements. Due to the lipophilic binding of P34 to oil bodies, hydrophobic interaction chromatography (HIC) was tested subsequently as a possible, alternative mode of interaction.

**Figure 3:**
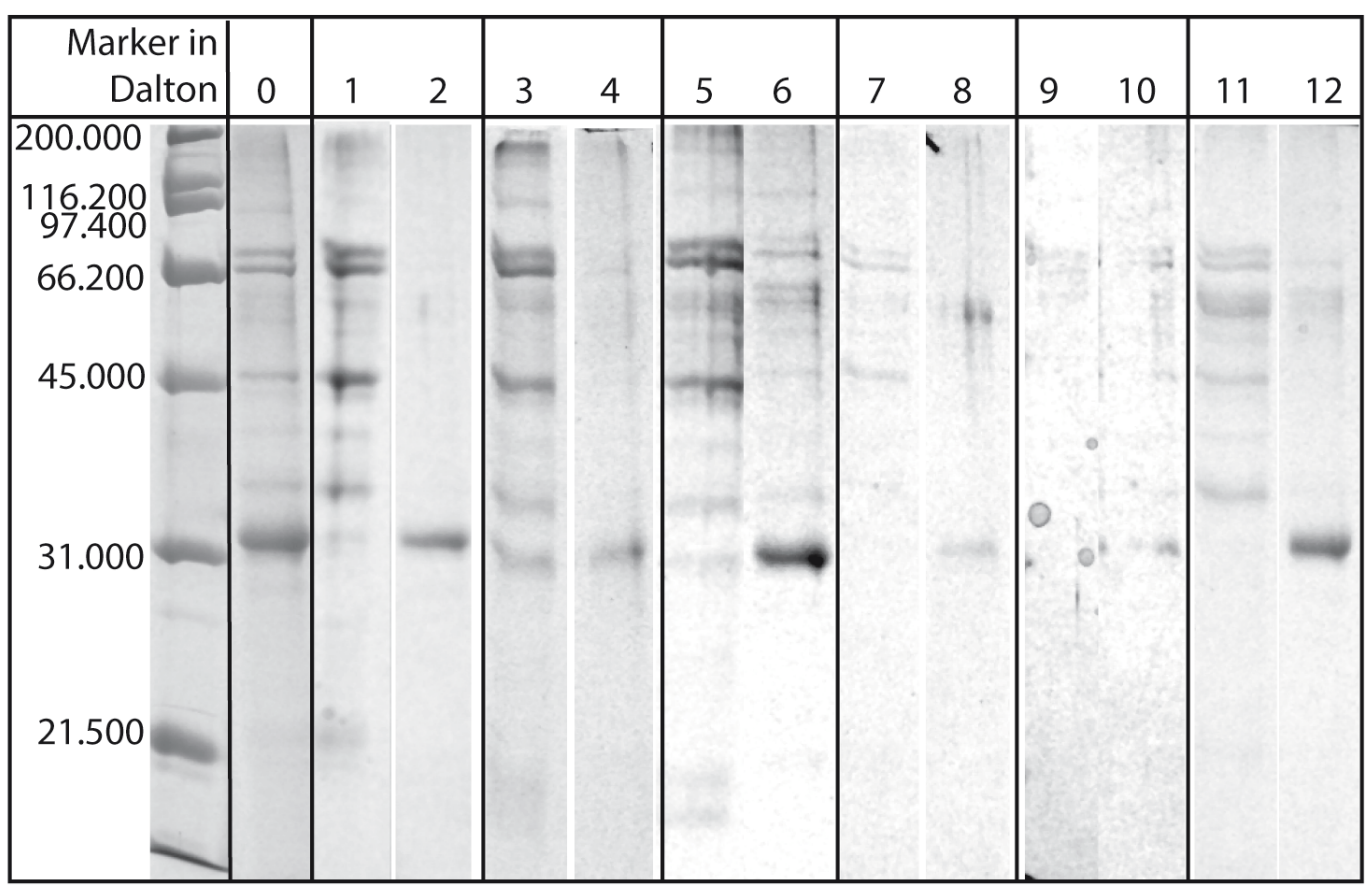
**Gel-electrophoretic analysis of flow-through and elution for six differently functionalised HIC resins**. Fractions from different resins, as described in the Methods section, were precipitated and separated using gel electrophoresis; from each column, two lanes (eluted proteins in binding buffer (flow-through) and at 0.4 M (NH_4_)_2_SO_4_) of every gel were included into this figure. P34 is represented by a band at ~32 kDa. Lane 0 represents the soybean feedstock used as column feed (diluted to 1 μg of total protein), lanes 1 and 2 are eluted fractions collected from Butyl Sepharose 4 Fast Flow, lanes 3 and 4 from Butyl-S Sepharose 6 Fast Flow, lane 5 und 6 from Octyl Sepharose 4 Fast Flow, lane 7 and 8 from Phenyl Sepharose 6 Fast Flow (low sub), lane 9 and 10 from Phenyl Sepharose 6 Fast Flow (high sub) and 11 and 12 from Phenyl Sepharose High Performance.

In order to select an appropriate stationary phase, six differently functionalised HIC resins were tested as described in the methods section. For every resin, samples were collected during the whole elution process. For the sake of brevity, only the results of the electrophoretic analysis of flow-through at 1 M (NH_4_)_2_SO_4 _(first lane for every resin), containing unbound proteins, and of elution at 0.4 M (NH_4_)_2_SO_4 _(second lane for every resin) are given in Fig. 3. The initial feedstock, diluted to 1 μg of total protein, is given in lane 0 as reference. As it can be deduced from a comparison of lanes 1, 3, 5, 7, 9 and 11, the chosen conditions for the binding buffer were high enough to promote the adsorption of P34 (band at ~32 kDa), but not the adsorption of a large number of contaminants. This is beneficial in two ways. Obviously, it simplifies purification, as non-bound components do not disturb the subsequent separation. Furthermore, it can increase the effective capacity of the resin for P34, as the target protein has to compete for adsorption sites with less other components. Using the elution steps described in the Methods section, it was possible to elute P34 by gradually decreasing salt concentration. Except for Phenyl Sepharose 6 Fast Flow (low sub) and Phenyl Sepharose 6 Fast Flow (high sub), P34 could be eluted at 0.4 M (NH_4_)_2_SO_4_. On the other two resins, P34 adsorbed stronger and only a very small amount of P34 could be eluted at this salt strength (lanes 8, 10), together with other contaminants. The majority of P34 was found at 0 M (NH_4_)_2_SO_4_, again together with a large number of contaminants (not shown). An optical comparison of lanes 2, 4, 6, 8, 10 and 12 revealed that the best purity for P34 could be achieved with Butyl Sepharose 4 FF. With this resin, almost no P34 was found in the flow-through and only a small amount of low-molecular contaminants co-eluted with the target protein. Consequently, Butyl Sepharose 4 FF was chosen as stationary phase for the preparative purification of P34. For the selected material, gradient elution conditions were examined in more detail, using additional step gradient experiments with HiTrap 1 mL resins to select appropriate salt concentrations for washing and elution steps. Those were performed using a chromatographic apparatus. It was found that a two step gradient with elution steps of 0.6 M (NH_4_)_2_SO_4 _and 0.4 M (NH_4_)_2_SO_4 _and a 1 M (NH_4_)_2_SO_4 _binding buffer gave satisfying results. As in the screening experiments, P34 was obtained during elution with 0.4 M salt. However, due to the small column size of 1 mL and the resulting small capacity, the amount of produced protein was not sufficient for allergologic studies. Therefore, the column size was increased.

### Scale-up of purification 1: From 1 mL to 7.5 mL

**Figure 4:**
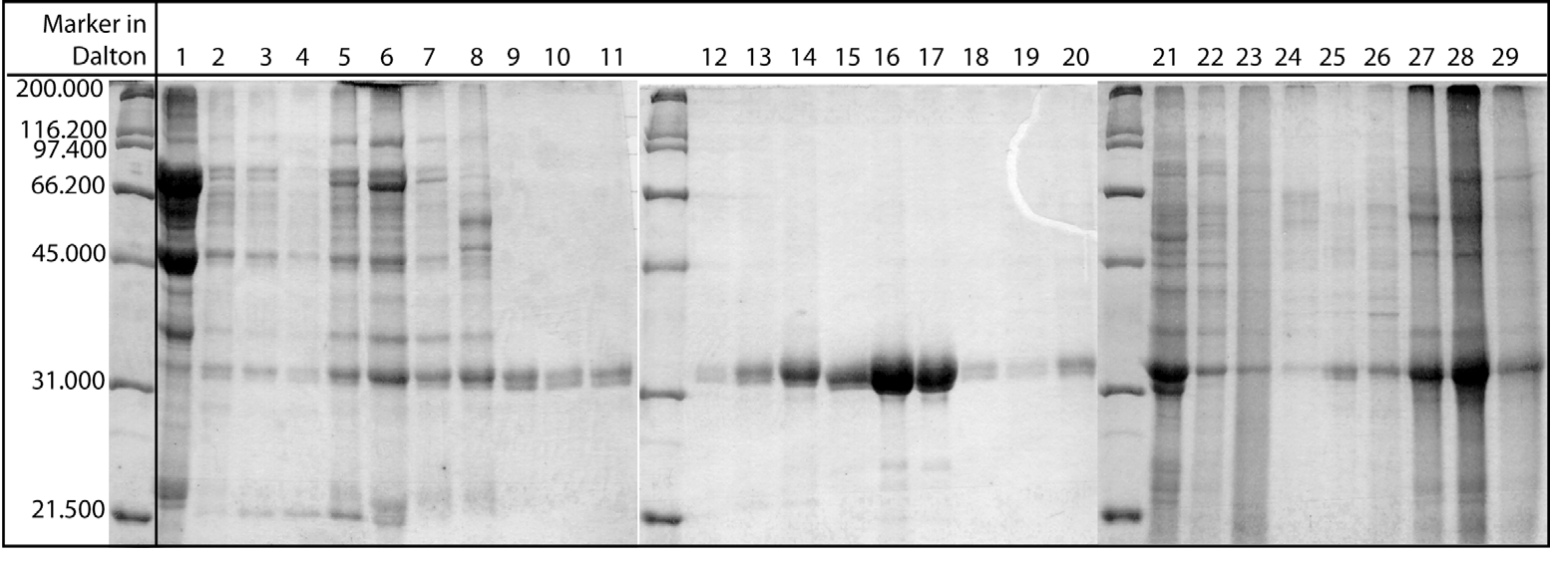
**Gel-electrophoretic analysis of a two-step elution in column 1**. Column 1 was loaded with soybean feedstock mixture (705 μg total protein, 77% of which are P34) in initial binding buffer (1 M (NH_4_)_2_SO_4 _in 0.05 M phosphate buffer, pH 6.5). Elution was performed at 0.6 M and 0.4 M (NH_4_)_2_SO_4_. In the Coomassie-stained polyacrylamide gel, one fraction is represented by one lane: lanes 1 – 4 show the flow-through in binding buffer, lanes 5 – 12 the fractions eluted with 0.6 M (NH_4_)_2_SO_4 _and lanes 13 – 21 the fractions eluted with 0.4 M (NH_4_)_2_SO_4 _and lanes 22 29 represent the fractions eluted in phosphate buffer during the washing step.

As described in the Methods section, 7.5 mL of Butyl Sepharose 4 FF bulk material, packed into column 1 (10 × 150 mm), were used as a first scale-up step. The column was loaded with the feedstock protein mixture, containing 705 μg of total protein, 77% of which are P34, in the binding buffer. P34 could be eluted from the column during the second elution step (0.4 M (NH_4_)_2_SO_4_). During separation, fractions were collected and analysed. The results of the SDS-PAGE are shown in Fig. 4. There, lanes 1–4 show the flow-through, in which unbound proteins can be found. The measurement of the protein concentration in every fraction using the Bradford method [19] showed that these amounted to 19% of total protein loaded on the column. By far the largest percentage represented contaminants, which, due to the chosen initial salt conditions, did not bind to the column and could be removed during loading. Only a very small amount of P34 (band at ~32 kDa) could be found in the flow-through. This indicates that the column capacity was reached with the loaded amount of protein. With the first elution step (0.6 M (NH_4_)_2_SO_4_), nearly all of the weakly bound proteins were removed (lanes 5–12), representing 12% of total protein content. Again, a small amount of P34 co-eluted during this step. A possible reason for this behavior could be different interaction sites on the protein surface, differing either in hydrophobicity or in steric availability. If a second adsorption site is less hydrophobic, a protein interacting with this site will elute at a higher salt level. Also, certain structures in the proximity of the adsorption site on the protein surface might interfere sterically with the stationary phase, thereby also weakening the adsorption. However, a detailed analysis would require a large effort, and since the amount of P34 eluting early is very small, it was not studied further in this body of work.

**Figure 5:**
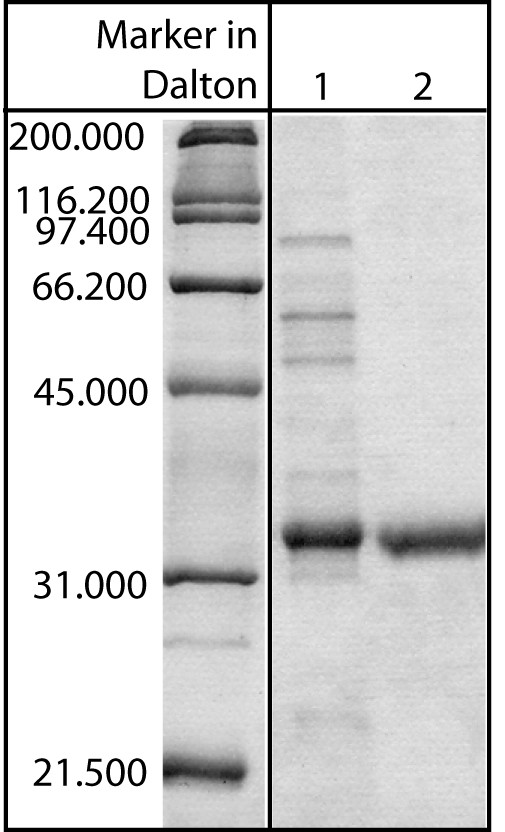
**Electrophoretic analysis of eluted protein P34 after dialysis**. In this Coomassie-stained polyacrylamide gel, lane 1 shows the soybean feedstock before separation and lane 2 the final, purified P34 fraction. 2 μg were loaded onto each lane.

In the second elution step at 0.4 M (NH_4_)_2_SO_4_, (lanes 13–17, Fig. 4), it was possible to elute and obtain highly pure P34. The protein content in these five fractions corresponded to approximately 18% of the P34 in the feedstock mixture and to 14% of the total protein found. Strongly bound proteins were eluted without ammonium sulphate within fraction 22 to 29, representing around 47% of total protein. As it can be seen in the gel, a certain amount of P34 is lost during this washing step. This could again be ascribed to different interaction sites or numbers of interactions between the protein and the ligands on the stationary phase. However, a lower second elution step could also result in co-eluting further contaminants. This aspect will be studied in more detail later in this contribution. Dialysing the collected P34 fractions with membranes exhibiting a molecular cut-off of 6–8 kDa during sample preparation for electrophoretic analysis seemed to further increase purity up to approximately 99% (determined using SDS-PAGE, Fig. 5 lane 2), as the traces of low molecular weight contaminations still present in the elution fractions (comp. lanes 16 and 17, Fig. 4) were also removed. The success of the separation in terms of achieved product purity can be seen nicely by comparing the soybean feedstock and the final, purified fraction (Fig. 5). A discussion of process yield follows later in this contribution. The results described could be reproduced more than seven times without significant deviations.

### Scale-up of purification 2: From 7.5 mL to 75 mL

**Figure 6:**
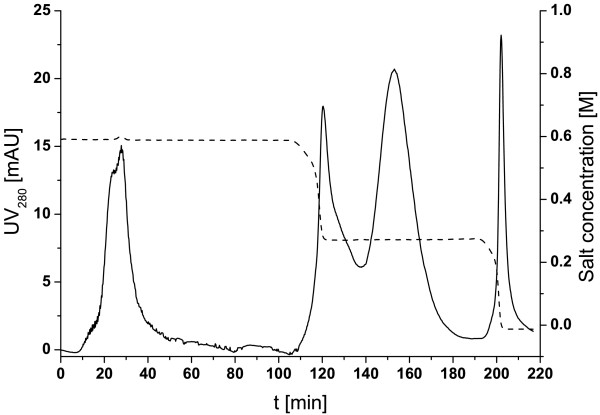
**Chromatogram of one-step gradient elution using column 2**. In this separation, 13 mg of protein were loaded onto the 75 mL column. Binding buffer contained 0.6 M (NH_4_)_2_SO_4_. Protein P34 was eluted using 0.25 M (NH_4_)_2_SO_4_. Subsequently, still bound protein at the column was eluted with phosphate buffer containing no (NH_4_)_2_SO_4_.

**Figure 7:**
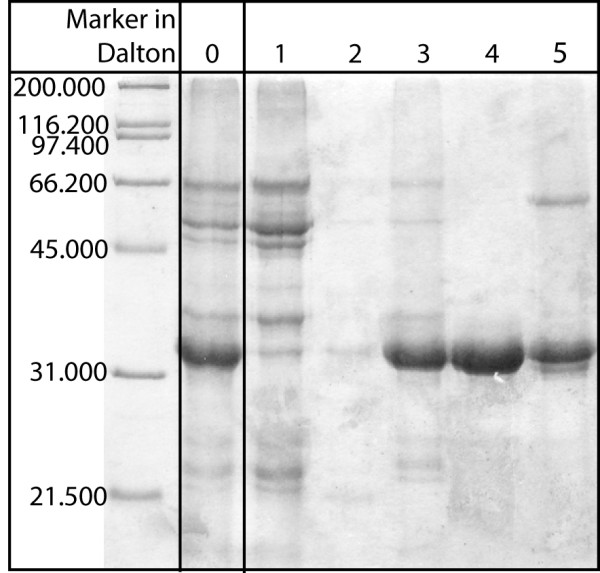
**Electrophoretic analysis of a one-step elution using column 2 (stored column feed)**. Fractions of a one-step separation, performed on the 75 mL column, were analysed. The fractions were concentrated and equal amounts were loaded on the shown Coomassie-stained gel as described in the Methods section. Lane 0 shows the column feed (soybean feedstock), lanes 1 and 2 the flow-through in binding buffer (0.6 M (NH_4_)_2_SO_4_), lanes 3 and 4 two peaks during the elution step with 0.25 M (NH_4_)_2_SO_4 _(comp. Fig. 6) and lane 5 the proteins eluted without (NH_4_)_2_SO_4 _in 0.05 M phosphate buffer.

In a subsequent step, the column volume was increased further to 75 mL of Butyl Sepharose 4 FF using column 2 (25 × 250 mm). The same two elution steps were applied at the beginning. With those, pure protein could be obtained. However, a more detailed analysis of the proteins eluting with the washing steps showed that at the beginning of the washing step, still pure P34 eluted from the column. This indicated the need for further optimisation. Therefore, different combinations of concentrations for binding and elution buffer were tested. A concentration of 0.6 M (NH_4_)_2_SO_4 _during loading was found to be best suited, as P34 already started to elute at lower concentrations. As the experiments with the smaller column 1 have already shown, after elution with 0.4 M (NH_4_)_2_SO_4_, there are still contaminating proteins adsorbed to the column (Fig. 4, lanes 27–29), prohibiting an elution at 0 M salt. Further experiments for determining an optimal elution concentration then showed that with an elution step at 0.25 M (NH_4_)_2_SO_4_, P34 could be eluted pure with an increased recovery yield when compared to elution at 0.4 M (NH_4_)_2_SO_4_. In order to shorten process time, it was further tested, whether the two step elution design with 0.6 (or 0.4) M (NH_4_)_2_SO_4 _and 0.25 M (NH_4_)_2_SO_4 _and 1 M (or 0.6) (NH_4_)_2_SO_4 _as binding buffer could be simplified to a one step elution process using 0.6 M salt in the binding buffer and 0.25 M salt for elution. A chromatogram of a one-step separation is given in Fig. 6. During elution, two overlapping peaks were observed. For a detailed analysis of protein elution sequence, both peaks were fractionised and analysed electrophoretically. The results are given in Fig. 7. It can be seen that some P34 eluted already during the first elution peak (lane 3), together with contaminants, but the majority of the target protein could be obtained pure in the second elution peak (lane 4). As indicated by the band in lane 5, P34 again could not be eluted completely from the column, but since the focus in this contribution was on maximizing purity, not yield, this one-step elution process could be considered successful. These results could also be reproduced successfully.

**Figure 8:**
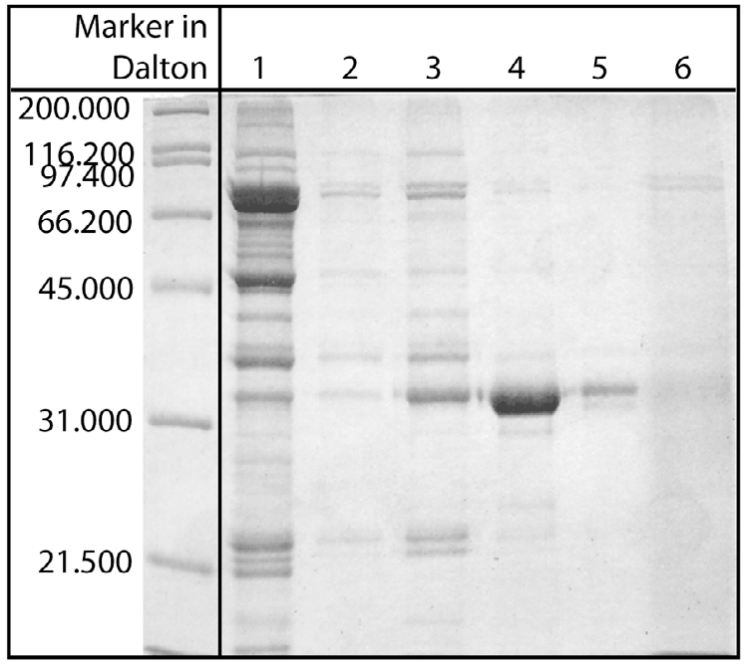
**Electrophoretic analysis of a one-step elution using column 2 (fresh column feed)**. Fractions of the one-step separation performed on the 75 mL column were analysed as described in the Methods section. Lanes 1 and 2 represent the flow-through in binding buffer (0.6 M (NH_4_)_2_SO_4_), lanes 3 and 4 the two elution peaks at 0.25 M (NH_4_)_2_SO_4 _and lanes 5 and 6 the proteins eluted without (NH_4_)_2_SO_4 _in 0.05 M phosphate buffer.

During one of the subsequent runs, however, using identical conditions, a slightly different elution pattern was found, even though the basic shape of the elution peaks was comparable to Fig. 6. Again, fractions were collected and analysed electrophoretically (Fig. 8). Here, lanes 1–2 represent the fractions collected in the flow-through, lanes 3 and 4 the two elution peaks and lanes 5 and 6 the fractions collected during the washing step. It can be seen that the fraction of P34 eluting already at the beginning of the step (lane 3), along with a large number of contaminants, seems to be smaller than in Fig. 7. The second peak still contained by far most of the P34 at very high purity, but the amount of contaminants was slightly larger than in Fig. 7. Additionally, a new band directly below P34 was found (lane 4, Fig. 8). It has to be noted that this additional band in the second elution peak could not be found in earlier runs with either one-step (Fig. 7) or two-step elution (compare Fig. 3, lane 6). A possible explanation for this type of behaviour is a difference in storage time. The homogenate used for earlier studies was stored in a refrigerator up to a few weeks, while the homogenate used for the experiment in Fig. 8 was prepared immediately before separation. It could be suspected that a remaining proteolytic activity in the stored protein suspension led to a partial digestion of some proteins, which might influence their binding behaviour, thereby simplifying separation. Still, even in this worst case, P34 purity was sufficiently high. Using this one-step approach, separation of the soybean feedstock could be achieved within 200 minutes. In comparison to the method published by Ogawa et al. [4], the approach described above is faster and considerably easier to realise.

### Estimation of yields for protein P34

In order to compare process performance in terms of yields, it has to be distinguished between total process efficiency, including the preparation of the soybean feedstock according to [11] (see Fig. 1) and starting with the soybean itself, and the efficiency of HIC purification as a stand-alone operation, starting with the prepared soybean feedstock.

For evaluation of total process performance, the amount of pure P34 obtained has to be related to the P34 content in the soybeans used. As stated already in the Background section, previous studies have shown that P34 represents 2–3% of total soybean protein [7]. Based on the manufacturer s specifications, 39% of the cultivars total mass are proteins, leading to a theoretical maximum amount of 7.8 mg of pure P34 per gram soybean for a P34 content of the total soybean protein of 2%. This also gives the highest possible yield to be obtained during purification. It has to be stressed that these values are only estimates, as both the P34 content as well as the protein mass percentage of the total soybean mass might vary in the beans used for this study. However, these numbers are useful to give at least some measure for the yield's order of magnitude for this type of process. For the case of the 7.5 mL column, where a solution extracted from 1 g soybeans was used, the best result obtained was 173 μg of pure P34. This corresponds to a total yield of ~2%. For column 2 (75 mL) using both two-step and one-step elution, an average amount of 250 μg of pure P34 could be obtained per gram soybean, increasing the overall yield slightly to ~3%. At first glance, this low yield seems disappointing. However, a more detailed analysis of P34 concentrations in the course of separation showed that the majority of protein loss occurred already during feedstock preparation. From 200 g soybeans only 192 mg of P34 could be found in the soybean feedstock used for chromatography, resulting in a yield of only 12%. A modified preparation protocol could help to increase the total process performance significantly.

Consequently, in order to evaluate the chromatographic performance itself, the amount of protein introduced with the column feed, the soybean feedstock has to be taken as reference, resulting in a yield of 18% for P34 for column 1 and 27% for column 2. This is comparable to other HIC processes reported for the isolation of a single protein from a multicomponent mixture [20], but still low when compared to other chromatographic techniques. For the present example, however, the relatively low yields are somewhat compensated by the low costs and high availability of soybeans.

### Confirmation of protein identity and properties

**Figure 9:**
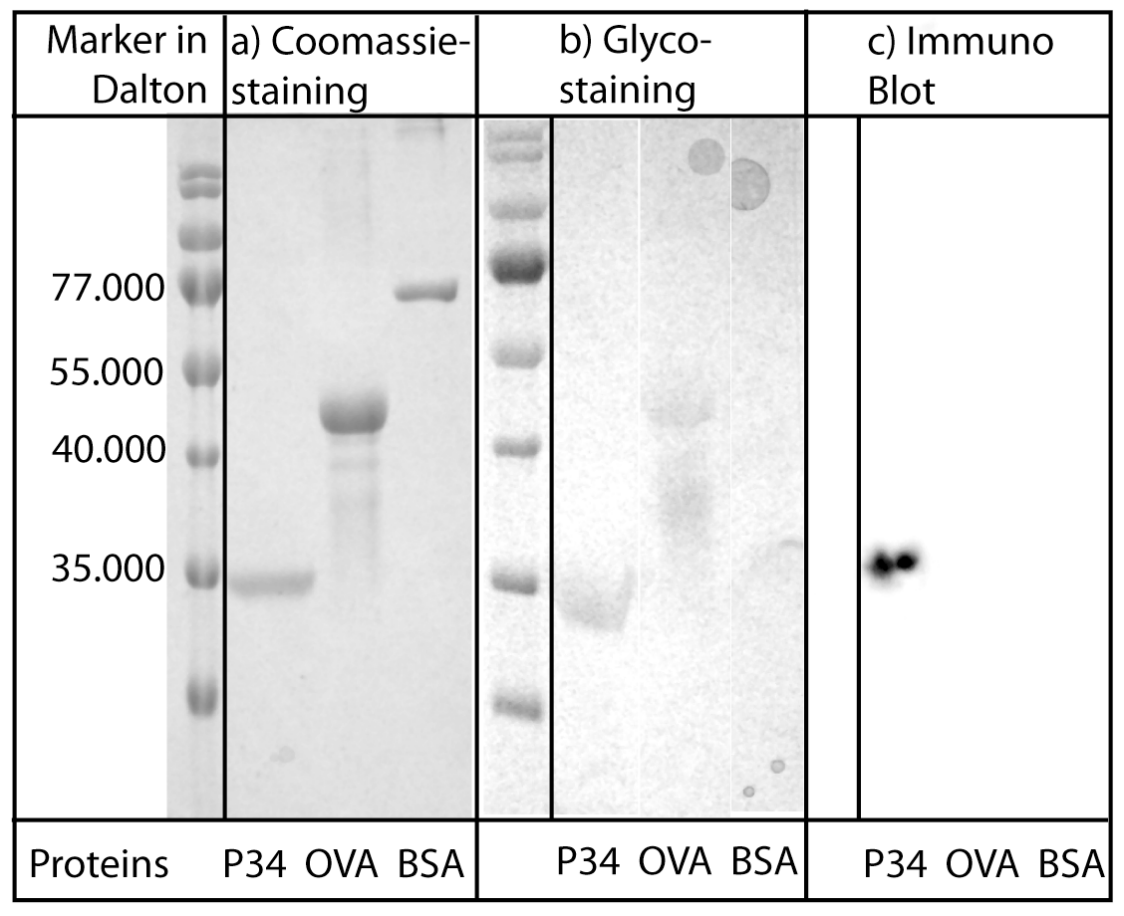
**Summary of electrophoretic analysis of protein properties**. P34 samples were collected during chromatographic purification and analysed using SDS-PAGE and different marking techniques (Coomassie-staining, proteins treated with Schiffs reagent and Immuno Blot). 9a: The Coomassie-stained proteins P34, Ovalbumin, BSA are shown (2 μg each lane). 9b: Proteins P34, Ovalbumin, BSA are shown treated with Schiffs reagent (2 μg each lane). 9c: Proteins P34, Ovalbumin, BSA were blotted and immuno-detected with mAb F5.

To clearly identify protein P34, the corresponding band was cut out of a Coomassie stained SDS-PAGE gel and analysed by in-gel digestion and mass spectrometry. A database analysis using MASCOT software and MSDB and/or NCBI protein databases identified the protein band as *Glycine max *(soybean) protein P34, also called Gly m Bd 30 K or Gly m 1. Glycosylation of P34 was tested as described in the Methods section. A Coomassie-stained gel, containing the purified P34 as well as the control proteins BSA and ovalbumin, is also given as reference in Fig. 9a. P34 was positive for the glycosylation staining and gave a violet band (Fig. 9b grey bands), when compared to BSA (negative for the staining, no band) and Ovalbumin (glycoprotein, grey band). This result indicates the presence of a glycosylation. Another indicator for constant glycosylation and size is the comparable electrophoretic behaviour of the obtained protein P34 in all SDS-PAGE analyses done in the course of this study. Thus, it can be concluded that glycosylation of P34 has not been changed significantly during separation. P34 was also successfully immunodetected with the monoclonal antibody F5, binding to amino acids 115–132. [21]. The result of this procedure is given in Fig. 9c.

## Conclusion

Based on a well-known purification protocol used for feedstock preparation [11], an effective and easy method for isolating the important allergen P34 using hydrophobic interaction chromatography was presented. A broad screening study led to the selection of Butyl Sepharose 4 FF as suitable stationary phase. For this resin, appropriate (NH_4_)_2_SO_4 _concentrations for binding and for two-step elution were determined experimentally using prepacked 1 mL columns. Based on these results, the process was scaled up twice to 7.5 and 75 mL of column volume, respectively. It was found that the conditions obtained with the small screening columns could be transferred to the larger columns satisfactorily. However, performance could be increased further, after several empiric optimisation steps were employed to obtain operating conditions for at first a two-step and secondly a one-step gradient elution process. Using one-step elution, process time could be reduced while the yield remained constant.

When considering total process yield, from the P34 content in the beans to the amount recovered in the final, purified solution, only 3% of available P34 were recovered. A systematic analysis of protein concentrations during the complete process showed that almost 88% of P34 were lost during the feedstock preparation process, indicating a need for process optimisation even before chromatography. When relating the amount of obtained, pure P34 to the P34 concentration in the feedstock, the HIC process itself performed comparable to other reported HIC applications, resulting in a yield of 27%. Due to the empirical optimisation, separation performance can probably be increased further using a model-based approach.

With the method presented was possible to obtain a sufficient amount of pure P34 in a simple way, compared to the possible alternatives. The use of step changes in gradient conditions allows the application of a simple column-syringe set-up.

## Methods

### Materials and equipment

All chemicals and biochemicals, unless indicated otherwise, were purchased from Roth (Karlsruhe, Germany) and were of analytical grade. Ovalbumin was purchased from Sigma Aldrich (Steinheim, Germany). Dried soybeans (according to manufacturer s specifications cultivars Heinong 40 und Dongnong 42) were purchased from Hensel Soja-Kost (Magstadt, Germany). Centrifugation was carried out in an Avanti J-25 High Performance centrifuge (Beckman Coulter, Krefeld, Germany). Prepacked HiTrap-columns (1 mL, 7 × 25 mm) of Phenyl Sepharose High Performance, Phenyl Sepharose 6 Fast Flow (low sub), Phenyl Sepharose 6 Fast Flow (high sub), Butyl-S Sepharose 6 Fast Flow, Butyl Sepharose 4 Fast Flow (FF) and Octyl Sepharose 4 Fast Flow as well as Butyl Sepharose 4 Fast Flow as bulk material (Lot number: for 7.5 mL column 10000941, for 75 mL column 10003753) were purchased from GE Healthcare (Uppsala, Sweden). Both a Merck Superformance column (10 × 150 mm, Merck, Darmstadt, Germany) and an Omnifit glass column (25 × 250 mm, Omnifit, Cambridge, U.K.) were used. The smaller column is referred to as column 1, the larger as column 2.

Chromatographic screening experiments were performed using either a syringe or an ÄKTA prime unit (GE Healthcare, Uppsala, Sweden). Preparative separations were carried out in the ÄKTA prime unit. As binding buffer 1 M (NH_4_)_2_SO_4 _in 0.05 M phosphate buffer (Na_2_HPO_4 _and NaH_2_PO_4_) at pH 6.5 was used. Buffer solutions were prepared using high purity water from a Millipore System (EASYpure RF, Barnstead, Germany). Fractions were concentrated using centrifugal devices with a molecular cut-off of 3 kDa (Roth, Karlsruhe, Germany). All percentages are given as volume percent unless stated otherwise.

### Preparation of soybean protein solution

Soybean solution was prepared according to the methods described in [8,11]. The feedstock preparation procedure was shown schematically already in Fig. 1. The soybeans were soaked overnight in distilled water, homogenised in 0.1 M Tris-HCl (pH 8.6) using a hand blender and pushed through a layer of gaze. The homogenate which passes the gaze was centrifuged for 20 min (53000 × g, 4°C) (Fig. 1, steps 1–3). The recovered oil body again was washed in 0.1 M Tris-HCl (pH 8.6) and centrifuged. Afterwards, the oil body was homogenised twice in 0.1 M Tris-HCl, 0.5 M NaCl (pH 8.6) and centrifuged (Fig. 1, step 4). To recover the protein, the oil body was finally homogenised in 0.1 M sodium carbonate and centrifuged as described before. The supernatant was concentrated in PEG (20 g/100 mL) using dialysis membranes and dialysed into the binding buffer afterwards (Fig. 1, step 5).

### Screening of hydrophobic interaction resins

Each column was equilibrated with binding buffer before 200 μL of protein solution (1 g/L) were applied to the column with a syringe. After washing the column with 15 column volumes (cv) of binding buffer, proteins were eluted with a successive multi-step gradient of 9 cv of 0.8 M, 0.6 M, 0.4 M, 0.2 M and 0.0 M (NH_4_)_2_SO_4_, respectively, in 0.05 M phosphate buffer at pH 6.5.

The collected fractions of 1.5 mL were precipitated with trichloric acid (10 g/100 mL), the pellets washed with acetone and analysed using SDS-PAGE (10% gels) and Coomassie-staining.

### Chromatographic separation and scale-up

Two different types of glass columns were used in this study. In column 1, 7.5 mL of Butyl Sepharose 4 FF Sepharose were packed hydrodynamically. Before separation, the column was equilibrated with at least 10 cv of binding buffer. Subsequently, 500 μl of soybean feedstock, containing 705 μg of total protein, of which 541 μg were P34, were loaded onto the column using a sample loop. For P34 separation, a two step gradient of 0.6 M (NH_4_)_2_SO_4 _and 0.4 M (NH_4_)_2_SO_4 _as well as a washing step at 0.0 M (NH_4_)_2_SO_4 _in 0.05 M phosphate buffer (pH 6.5) was used. Each gradient step was held until a constant UV signal could be observed. After each run, the column was washed with a minimum of 10 cv of 0.05 M phosphate buffer and a minimum of 10 cv of 20% ethanol to remove strongly bound contaminants.

In a subsequent experiment, column 2 was packed with 75 mL Butyl Sepharose 4 FF Sepharose. Before separation, it was equilibrated with 4 cv of binding buffer (0.6 M (NH_4_)_2_SO_4 _in 0.05 M phosphate buffer, pH 6.5), before 5 mL of protein solution in binding buffer, containing at least 13 mg of total protein, 77% of which are P34, were applied to the column with a sample loop. Proteins were eluted using either a two or a one step gradient of 0.4 M (NH_4_)_2_SO_4 _and 0.25 M (NH_4_)_2_SO_4 _or only 0.25 M (NH_4_)_2_SO_4 _in 0.05 M phosphate buffer (pH 6.5). Each gradient step was held until a constant UV signal was observed. The same cleaning procedure was used as described for column 1.

### Electrophoretic analysis of eluted fractions

For the analysis of the fractions for Fig. 4, 10 mL (fractions for lane 1–19) to 15 mL (fractions for lane 20–29), collected during chromatography with column 1 for subsequent analysis, were concentrated to ~30 μL and analysed using SDS-PAGE (10% gels) and Coomassie-staining. A Bradford assay with 2–10 μg BSA-standards was used to determine total protein concentration in each fraction [19]. For Fig. 7 and 8, 4 mL of each fraction were concentrated and analysed using SDS-PAGE (10% gels) and Coomassie-staining. Densitometric analysis of gels was conducted using an AlphaEase FC Imaging System (Alpha Innotech Corp., San Leandro, CA., U.S.A.).

### Detection of glycosylation

After electrophoresis, the separated proteins were fixed in the gel using 70% methanol and 10% acetic acid for 30 minutes. Afterwards, the gel was washed in water for 5 minutes. Subsequently, the gel was incubated with periodic acid (1 g/100 mL)/30% acetic acid for 30 minutes and treated with Schiffs reagent over night. All glycosylated proteins give violet bands [22]. P34 was compared to the reference proteins BSA (no glycosylation) and ovalbumin (glycosylation).

### Immunodetection of P34

For immunodetection of P34 with monoclonal antibody F5 (kindly provided by T. Ogawa, Koyoto University, Osaka, Japan), protein samples were separated electrophoretically on 10% SDS-polyacrylamide gels, and blotted semi-dry onto nitrocellulose membranes. The membrane was then stained with F5 and a BM Chemiluminescence Western Blotting Kit (mouse/rabbit, Roche, Grenzach-Wyhlen, Germany) following the manufacturer s instructions. Finally, the blots were analysed on an Alpha Ease FC Imaging System.

## Authors' contributions

ES performed pre-purification, syringe experiments and subsequent electrophoretic analysis as well as column chromatography. LCK conceived of the chromatographic process, and participated in column chromatography as well as data analysis. Both authors contributed equally to writing and revising the article draft. ASM and HJR initiated the study, participated in study design and critical revision of the manuscript.
